# Sleep Disturbance Alters Cocaine-Induced Locomotor Activity: Involvement of Striatal Neuroimmune and Dopamine Signaling

**DOI:** 10.3390/biomedicines10051161

**Published:** 2022-05-18

**Authors:** Soheil Kazemi Roodsari, Yan Cheng, Kirstin M. Reed, Laurie L. Wellman, Larry D. Sanford, Woong-Ki Kim, Ming-Lei Guo

**Affiliations:** 1 Drug Addiction Laboratory, Department of Pathology and Anatomy, Eastern Virginia Medical School, Norfolk, VA 23507, USA; roodsask@evms.edu (S.K.R.); chengy@evms.edu (Y.C.); reedkm@evms.edu (K.M.R.); 2 Center for Integrative Neuroscience and Inflammatory Diseases, Eastern Virginia Medical School, Norfolk, VA 23507, USA; wellmall@evms.edu (L.L.W.); sanforld@evms.edu (L.D.S.); kimw@evms.edu (W.-K.K.); 3 Sleep Research Laboratory, Department of Pathology and Anatomy, Eastern Virginia Medical School, Norfolk, VA 23507, USA; 4 Department of Microbiology and Molecular Cell Biology, Eastern Virginia Medical School, Norfolk, VA 23507, USA

**Keywords:** sleep fragmentation, cocaine, microglia, neuroinflammation, drug addiction, dopamine

## Abstract

Sleep disorders have high comorbidity with drug addiction and function as major risk factors for developing drug addiction. Recent studies have indicated that both sleep disturbance (SD) and abused drugs could activate microglia, and that increased neuroinflammation plays a critical role in the pathogenesis of both diseases. Whether microglia are involved in the contribution of chronic SDs to drug addiction has never been explored. In this study, we employed a mouse model of sleep fragmentation (SF) with cocaine treatment and examined their locomotor activities, as well as neuroinflammation levels and dopamine signaling in the striatum, to assess their interaction. We also included mice with, or without, SF that underwent cocaine withdrawal and challenge. Our results showed that SF significantly blunted cocaine-induced locomotor stimulation while having marginal effects on locomotor activity of mice with saline injections. Meanwhile, SF modulated the effects of cocaine on neuroimmune signaling in the striatum and in ex vivo isolated microglia. We did not observe differences in dopamine signaling in the striatum among treatment groups. In mice exposed to cocaine and later withdrawal, SF reduced locomotor sensitivity and also modulated neuroimmune and dopamine signaling in the striatum. Taken together, our results suggested that SF was capable of blunting cocaine-induced psychoactive effects through modulating neuroimmune and dopamine signaling. We hypothesize that SF could affect neuroimmune and dopamine signaling in the brain reward circuitry, which might mediate the linkage between sleep disorders and drug addiction.

## 1. Introduction

Sufficient restorative sleep is necessary for maintaining our physical and psychological health. However, chronic SDs, including insomnia, insufficient sleep time, and poor sleep quality, are highly prevalent in modern society and can arise from various social factors, including competition, sustained stress, and economic pressure, etc. [1], as well as a variety of health problems [2,3]. In the United States alone it has been estimated that nearly 25% of adults have sleep problems [4]. Chronic SDs also have high comorbidity with drug abuse, another worldwide public health concern [5], and are well-known as major risk factors in increasing the likelihood of drug addiction [6,7]. The neurological mechanisms underlying this comorbidity are unknown and remain mostly unexplored. However, sleep has extensive interactions with brain regions that are critical components of brain reward circuitry underlying addiction, including the striatum, prefrontal cortex, hippocampus, and amygdala [6,8,9]. These regions provide opportunities to understand the neural and mechanistic linkages between SDs and addiction. One of these mechanistic linkages may be neuroinflammation.

Drug addiction is now recognized as a neuroinflammation-related brain disease [10,11]. Psychostimulants, including cocaine, can activate microglia, both in vitro and in vivo [12,13], and increased microglial activation has been consistently found in various stages of cocaine addiction [14,15]. Microarray analyses of mouse brains have found cocaine- and methamphetamine-mediated upregulated expression of pro-inflammatory factors, such as IL1β, IL6, TNFα, and CCL2 in the prefrontal cortex and nucleus accumbens (NAc) [16,17], and cocaine self-administering macaques show a strong inflammatory response in the NAc [18]. Increased IL1β expression in the ventral tegmental area (VTA) is critical for cocaine-mediated behavioral changes, including conditional place preference (CPP) and self-administration [19]. In addition to animal work, the brains of drug addicts have revealed a close association between neuroinflammation and stimulant addiction with a significant increase in the number of activated microglia in chronic drug abusers [20,21]. Additionally, impaired microglial activation, due to toll-like receptor (TLR) 3 and 4 deficiency, blunted cocaine-mediated locomotion and CPP [22,23]. These findings underscore the role of neuroimmune pathways in the development of drug addiction, and targeting neuroimmune signaling has been suggested as a potential treatment avenue for preventing, or ameliorating, the development of drug addiction [24,25]. 

Sleep has an intimate reciprocal relationship with the immune system [26] and SDs are capable of inducing microglial activation and neuroinflammation, which, ultimately, lead to synaptic loss and neuronal dysfunction in various rodent models [27]. For example, sleep deprivation for 48 h in rats increased levels of IL1β, TNFα, IL6 and decreased anti-inflammatory factors Il4 and IL10 in the hippocampus, and led to spatial memory impairment [28], whereas microglia inhibition mitigated spatial memory loss [29]. Sleep restriction (20 h for 10 days consecutively) increased microglial Iba1 levels in the hippocampus in C57BL/6 mice [30]. Chronically sleep-restricted mice and rats show similar changes in neuroimmune signaling and neuronal dysfunction [31,32]. Interestingly, administration of minocycline, an inhibitor of microglial activation, is capable of blocking locomotor changes and self-administration behavior induced by abused drugs [33,34], and can also block some of the effects of sleep deprivation [35].

The role of microglia in addiction and the close association between sleep and the immune system suggest that microglia (mediators of neuroinflammation) might be a critical link between SDs and drug addiction. To test this hypothesis, we subjected mice to three weeks of SF, a common SD [36], with daily treatment of cocaine starting from two weeks after SF initiation, and assessed locomotor activity, neuroinflammation, microglial immune responses, and dopamine signaling in the striatum. Separate cohorts of mice were treated identically except that they received a cocaine challenge after one week of withdrawal and discontinuation of SF. Our results showed that ongoing SF significantly blunted cocaine-induced locomotion, mitigated neuroinflammation, and modulated microglial immune responses. There were no differences in the levels of postsynaptic density protein 95 (PSDs95), dopamine receptor 1 (DRD1), dopamine transport (DAT), and tyrosine hydroxylase (TH) in the striatum across the groups. In mice with cocaine withdrawal, prior SF decreased locomotor sensitivity that was induced by cocaine challenge and also modulated the effects of cocaine on neuroimmune and dopamine signaling in the striatum. These results suggest that SF could modulate microglial immune responses and dopamine signaling in the striatum and that such dysregulation might be an important link between sleep disturbance and drug addiction. 

## 2. Materials and Methods

### 2.1. Animal and Reagents

Wild type C57BL/6 mice (male, 8-week-old, 22–27 g on arrival) were obtained from Charles River Laboratories (Wilmington, MA, USA). They were housed and kept in a colony room with food and water available ad libitum. The colony room was maintained on a 12:12 light to dark cycle and ambient temperature of 24.0 °C ± 1.5 °C. All procedures were conducted in accordance with the National Institutes of Health’s Guide for the Care and Use of Experimental Animals and were approved by Eastern Virginia Medical School’s Institutional Animal Care and Use Committee (protocol number: 20-010, 09/2020). Cocaine was purchased from Millipore-Sigma (Product No. C5776, St. Louis, MO, USA) and was freshly dissolved in 0.9% saline solution for injections. 

### 2.2. SF Procedure and Cocaine Administration

SF was performed using commercial, validated devices (Lafayette Instruments, Sleep Fragmentation Chamber, model 80391) that employ an automated sweeper arm that moves across animal cages to disrupt sleep via tactile stimulation. In brief, the mice were separated into four groups receiving different treatments (±SF ± cocaine) and all animals were placed into the devices 1 day prior to the start of SF. For SF, sleep was interrupted at 2 min intervals during the 12 h light period (normal sleeping period of mice). This SF protocol in mice reportedly produces moderate to severe SF [37,38,39] without significantly reducing overall sleep or significantly impacting sleep macro- or micro-architecture [38,39]. During the 12 h dark period, the motorized mechanical sweeper was stopped and mice were free to behave normally. Animals were observed daily to assess their health and to assure proper functioning of the SF device. Sham animals were maintained in their home cages without any interruption. The mice were first subjected to two weeks of SF followed by one week of cocaine (15 or 10 mg/kg, *i.p.*) or saline administration. Such doses have been extensively employed in previous studies to explore the behavioral effects stimulated by cocaine [40,41] and also used to investigate the interaction between SDs and cocaine [42]. SF continued during the week of cocaine administration and the mice were subjected to locomotor tests (30 min) on every other day following cocaine injection. On the day after the last cocaine injection, the mice were subjected to open field and rotarod tests. SF and cocaine administration were discontinued in a separate cohort of mice. These animals received cocaine injection (10 mg/kg, *i.p.*) after one week of withdrawal, followed immediately by locomotor testing. 

### 2.3. Behavioral Experiments

(1) Locomotor analysis: On days 1, 3, 5, 7 of cocaine administration, immediately after injection, the mice were placed into an open field apparatus (Truescan, Coulbourn instrument, Allentown, PA, USA) to detect locomotor activity for 30 min. The TruScan system consists of a clear arena that records activity via photobeam breakages with 16 infrared sensors located at 1-inch intervals on a ring 3 cm above floor level. Data, including total horizontal distance, average velocity, and immobility time (%), were automatically collected and relayed to a c PC and interpreted by software. (2) Open field test (OFT): On the day after the last cocaine injection, the mice were put into the open field to test anxiety-related behaviors. The OFT started by placing a mouse in the corner of the apparatus and letting it move freely for 10 min. At the end of the test, the animal was returned to its home cage and the box was cleaned with 70% ethanol. The total distance travelled and the ratio of time (%) spent in the central area were measured and compared among groups. (3) Rotarod analysis: Before testing, the mice were trained for two days to learn how to balance on the rotarod (Bioseb Company, Pinellas Park, FL, USA) at a speed of 4 rpm. On the test day, the mice were put on the rotarod which was set to accelerate from 4 to 40 rpm over 5 min. Three trials were conducted and the average latency for each mouse to fall off the rotating shaft was recorded. The apparatus was cleaned with 70% ethanol between trials. Fall latencies were compared amongst groups of mice as a measure of locomotor coordination. 

### 2.4. Adult Microglia Isolation

Mice were anesthetized with 4% isoflurane and transcardially perfused with 1× PBS. The brains were then removed and quickly dissected into different regions including the striatum. The striatum was pooled from at least three mice for adult microglia isolation by using MACS dissociation kits (Miltenyi Biotech Company, San Diego, CA, USA), according to the recommended protocol. Briefly, around 500 mg of tissue was homogenized in 2 mL enzyme mixture by using a gentleMACS™ Octo Dissociator at 37 °C for 30 min. The homogenates were then applied to a MACS^®^ Smart Strainer (70 μM), followed by centrifuging at 300× *g* for 10 min at 4 °C. The acquired pellets were processed through debris and red blood cell removal procedures and dissolved in 500 μL labelling solution. The cells were then incubated with 15 μL CD11b beads for 15 min at 4 °C. followed by passing through a column in a magnetic field (positive selection). The purified microglia were then suspended in 1 mL 1× PBS with 0.5% BSA and quantified by countess 3 (Thermo Fisher). The purified adult microglia were seeded into 6-well plates for Ib1 immunostaining. The remaining striatal microglia were stored in a freezer at −0 °C for RNA extraction to determine levels of pro- and anti- inflammatory factors. 

### 2.5. RNA Extraction, Reverse Transcription, and Quantitative Polymerase Chain Reaction (qPCR)

Briefly, approximately 100 mg of brain tissue or 1 × 10^6^ adult microglia was directly added to 1 mL Trizol (Invitrogen). Brain lysates were briefly sonicated (3–5 s) and incubated for 10 min on ice and then aspirated into new 1.5 mL microcentrifuge tubes with 0.2 mL of chloroform added. After vigorous vortexing, the samples were centrifuged at 10,000× *g* for 15 min at 4 °C. The upper aqueous phase was transferred to a new tube and 500 μL isopropyl alcohol was added. Samples were then incubated for 10 min and centrifuged again to precipitate total RNA. The total RNA was dissolved in DEPC-treated H_2_O and quantified. Reverse transcription reactions were performed using a Verso cDNA kit (Invitrogen). The reaction system (20 μL) included 4 μL 5× cDNA synthesis buffer, 2 μL dNTP mix, 1 μL RNA primer, 1 μL RT enhancer, 1 μL Verso enzyme Mix (Invitrogen), total RNA template 500 ng, and a variable volume of water. Reaction conditions were set at 42 °C for 30 min. QPCRs were performed by using SuperScript™ III Platinum™ One-Step qRT-PCR Kit (Invitrogen). Reaction systems were set up as follows: 10 μL Master mix, 1.0 μL primers and probes, and 1 μL cDNA and 8 μL distilled H_2_O. 96-well plates were placed into a QS3 qPCR machine (Invitrogen) for program running. Mouse primers for TNFα, IL6, IL1β, CCL2, TGFβ, and IL10 were purchased from Invitrogen (Mm00443258, Mm00446190, Mm00434200, Mm00441242, Mm01227699, and Mm01288386, respectivley). Mouse GADPH (Invitrogen, Mm99999915) served as internal control for quantification. 

### 2.6. Western Blots

Brain tissues were dissolved in RIPA buffers with proteinase and phosphatase inhibitors (Thermo Scientific, Waltham, MA, USA) and sonicated for 10 s on ice at 70% amplitude (Thermo Scientific). The brain homogenates were then incubated at 4 °C for 30 min followed by 12,000 rpm centrifugation for 10 min. The supernatants were taken out and the protein concentrations were calculated through the BCA method. Equal amounts of the proteins (25 μg) were electrophoresed in a sodium dodecyl sulfate-polyacrylamide gel (160 V, 60 min) under reducing conditions followed by transfer to PVDF membranes (180 mA, 90 min). The blots were blocked with 3% nonfat dry milk in Tris-buffered saline (TBST). The Western blots were then incubated with indicated antibodies overnight at 4 °C. The next day, the membranes were washed and incubated with appropriate IRDye fluorescent mouse or rabbit second antibody for one hour at room temperature. After three washes with TBST, the membranes were put into the Odyssey^®^ Imaging System for image development and the intensity of fluorescent band were quantified using Image Studio™ Software. After imaging, the membranes were re-probed by β-actin for normalization. The following antibodies were used at the indicated concentration in our studies: microglial activation marker CD11b (1: 2000, NBP2-19019), astrocyte activation marker GFAP (1: 5000; Abcam, ab7260), NLRP3 (1:2000, adipogen, AG-20B-0014-C100), DRD1 (1: 2000, Proteintech, 17934-1-AP), DAT (1: 2000, Proteintech, 22524-1-AP), TH (1: 2000, Proteintech, 25859-1-AP), caspase 1 (1: 1000, Santa cruz, sc-56036), ASC (1: 1000, Santa cruz, sc-514414), β-actin (Santa Cruz; 1: 2000, sc-8432) or (Sigma; 1: 2000, A2066). Second antibodies were purchased from Li-COR company including IRDye^®^ 680RD Donkey anti-Mouse (1: 5000) or rabbit IgG; IRDye^®^ 800CW Donkey anti-Mouse or rabbit IgG (1: 5000). 

### 2.7. Double Immunofluorescence Staining

Mice were anesthetized with 4% isoflurane and transcardically perfused with 1× PBS followed fixed with 4% PFA. The brains were removed and put into 4% PFA overnight and washed with 1× PBS three times. The brains were embedded for section preparation (5 μM). Brain sections were co-incubated with primary anti-AIF1 antibody (1: 500, Wako Pure Chemical Industries, Osaka, Japan, 019-19741) or anti-GFAP antibody (1: 500, ab7260, abcam) overnight at 4 °C. Secondary AlexaFluor 488 goat anti-rabbit IgG (A-11008) or AlexaFluor 594 goat anti-mouse (A-11032) (Thermo Fisher Scientific Waltham, MA, USA) was added for 2 h to detect Iba1 and GFAP, followed by mounting of sections with prolong gold antifade reagent with 4,6-diamidino-2-phenylindole (Thermo Fisher Scientific, Waltham, MA, USA, P36935). Fluorescent images were acquired on a Zeiss Observer. Zenpro software (Carl Zeiss, Thornwood, NY, USA) and ImageJ was used to process and analyze the intensity Iba1 and GFAP signals. 

### 2.8. Statistical Analysis

All data are expressed as means ± the standard error of the mean (SEM). Data were statistically evaluated using one-way or two-way analyses of variance (ANOVA) procedures followed, when appropriate, by Tukey-Kramer multiple comparisons tests using GraphPad Prism 8 (La Jolla, CA, USA). Tests with probability levels of <0.05 were considered statistically significant. For behavioral tests, each group included at least five to eight mice for analysis. 

## 3. Results

### 3.1. Effects of SF on Cocaine-Mediated Behavioral Changes

**Figure 1:**
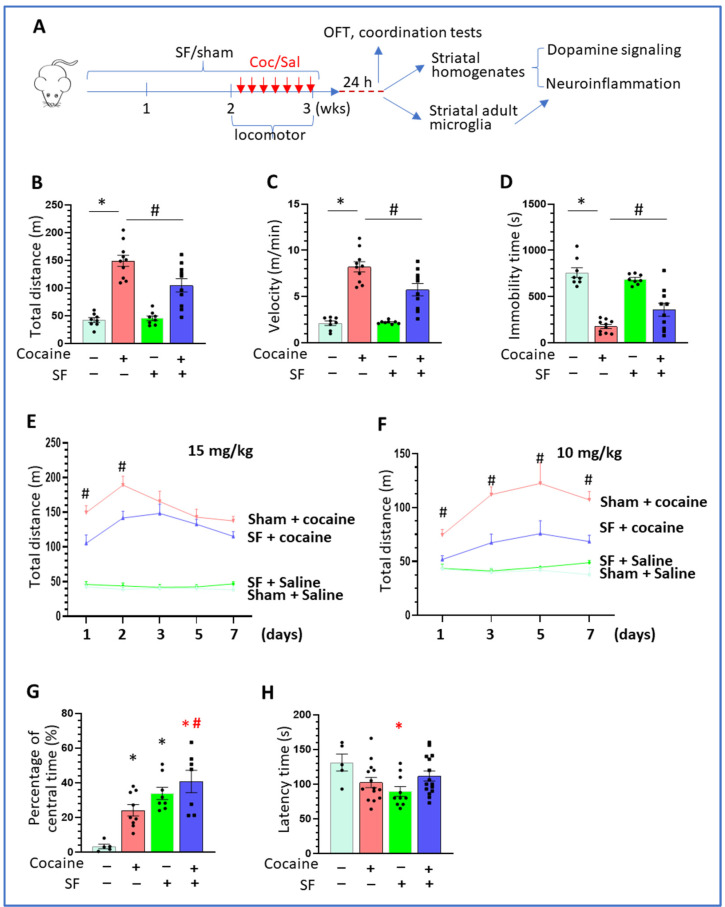
The Effects of SF on cocaine-mediated behavioral changes. (**A**) Schematic for the experimental design. (**B**) Cocaine significantly increased locomotor activity while SF partially blocked this hyperlocomotion (* *p* < 0.05, vs. sham + saline; # *p* < 0.05, SF + cocaine vs. sham + cocaine, n = 8–10). (**C**) Cocaine significantly increased the velocity while SF partially blocked this upregulation (* *p* < 0.05, vs. sham + saline; # *p* < 0.05, SF + cocaine vs. sham + cocaine, n = 8–10). (**D**) Cocaine significantly decreased the immobility time and SF partially blocked this downregulation (* *p* < 0.05, vs. sham + saline; # *p* < 0.05, SF + cocaine vs. sham + cocaine, n = 8–10). (**E**) SF decreased the distance travelled on the first and second day of cociane injection (15 mg/kg) during the 7-day injecition period (# *p* < 0.05, SF + cocaine vs. sham + cocaine). (**F**) SF decreased the distance travelled throughout the whole 7-day cocaine injection period (10 mg/kg) # *p* < 0.05, SF + cocaine vs. sham + cocaine). (**G**) Cocaine and SF increased the pecentage time in central area (* *p* < 0.05, vs. sham + saline; # *p* < 0.05, SF + cocaine vs. sham + cocaine, n = 8–10). (**H**) SF decreased the latency time of mice in the coordination test (* *p* < 0.05, vs. sham + saline).

Cocaine-induced hyperlocomotion, reflecting the excitability of striatal dopaminergic neurons, is one of the indexes of cocaine-mediated reward effects. Previous studies demonstrated that acute sleep deprivation could alter cocaine-mediated locomotion [42]. Here we explored the effects of SF on locomotor activity induced by cocaine (see Experimental Schematic in Figure 1A). As shown in Figure 1B, on the first day, total distances travelled were significantly different among groups [one-way ANOVA, F (3, 32) = 31.33, *p* < 0.001]. There was no significant difference between the sham + saline and SF + saline groups (*p* > 0.05). Cocaine significantly increased distance travelled in both sham + cocaine and SF + cocaine groups (*p* < 0.0001 and *p* = 0.002, respectively); however, the distance travelled of the SF + cocaine group was significantly lower than that of sham + cocaine group (*p* = 0.005). These data indicate that SF had no effects on the basal levels of locomotor activities but partially blocked the increase in activity induced by cocaine. There were also significant differences in movement velocity across groups (Figure 1C, one-way ANOVA, F (3, 32) = 35.36, *p* < 0.0001). SF significantly decreased cocaine-mediated upregulation of travel speed (*p* = 0.0043). SF also altered cocaine induced alterations in immobility time [Figure 1D, one-way ANOVA, F (3, 32) = 32.53, *p* < 0.0001]. Cocaine significantly reduced immobility time and SF could partially reverse the effects (*p* = 0.04). Taken together, these data demonstrate that SF could mitigate the psychoactive effects of acute cocaine administration. 

We next investigated whether SF altered cocaine-mediated effects over the course of 7 days of injections. There were significant differences in distance travelled (Figure 1E, two-way ANOVA; time x treatment: F (15, 152) = 3.287, *p* < 0.0001; time: F (3.689, 112.2) = 4.857, *p* = 0.0016; treatment: F (3, 34) = 81.69, *p* < 0.0001). Comparisons for SF + cocaine and sham + cocaine between the first and second day found significant differences in total distance (*p* < 0.005), whereas reductions in the average distance for the SF + cocaine group compared to the sham + cocaine did not reach significance on the latter days (*p* > 0.05). To confirm results that SF could mitigate the effects of cocaine on locomotor activity, we tested another batch of mice with two weeks of SF followed with one week of cocaine injections (10 mg/kg). This study also revealed significant differences in distance travelled (Figure 1F, two-way ANOVA analysis; time x treatment: F (9, 48) = 2.193, *p* = 0.039; time: F (1.824, 29.18) = 4.704, *p* = 0.0194; treatment: F (3, 16) = 34.64, *p* < 0.0001). There were significant differences in total distance travelled between the SF + cocaine and sham + cocaine groups beginning on the first day and continuing through the 7th day (*p* < 0.005). 

Chronic SDs may induce negative moods, such as irritation, anxiety, and depression, which are well-known factors that play critical roles in promoting craving and relapse of abusing drugs during the cycle of withdrawal [43,44,45]. One common test monitoring the depression- and anxiety- like behaviors is to determine the times spent in the central and peripheral area in the open field test (OPT) [46]. Therefore, we monitored the time (%) in the central area for each mouse one day after the last cocaine injection. Compared to the sham + saline group, SF and cocaine individually significantly increased the time that mice spent in the central area (Figure 1G, one-way ANOVA, F (3, 25) = 11.63, *p* < 0.001); there was also significant difference in times spent in the central area between the SF + cocaine and sham + cocaine groups (*p* = 0.03), indicating combined effects of SF and cocaine on depression-like behaviors. For rotarod tests examining locomotor coordination, only SF mice showed significantly decreased latency time period compared to sham + saline group (Figure 1H, one-way ANOVA, F (3, 40) = 3.063, *p* = 0.04) and there was no significant difference between the sham + cocaine and SF + cocaine groups (*p* > 0.05). In summary, our results revealed that SF could modulate cocaine-mediated behavioral changes, including reducing locomotor activity and enhancing depression-like behaviors.

### 3.2. SF Blunted Cocaine-Mediated Neuroinflammation in the Striatum

**Figure 2:**
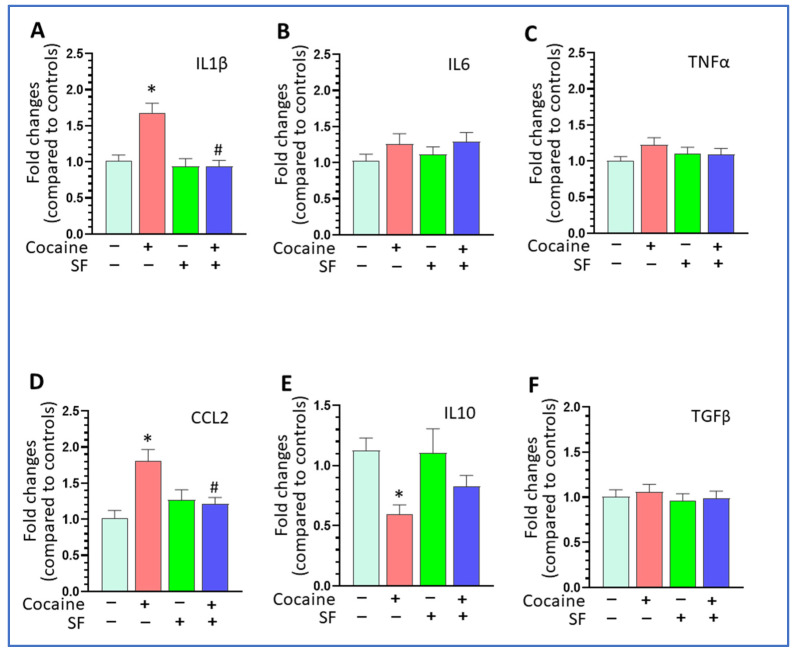
SF blunted cocaine-mediated neuroinflammation in the striatum. (**A**) Cocaine increased IL1β levels and SF blocked the increase (* *p* < 0.05, vs. sham + saline; # *p* < 0.05, SF + cocaine vs. sham + cocaine, n = 5). (**B**) There was no difference on IL6 levels across the four groups of mice (*p* = 0.3794). (**C**) There was no difference on TNFα levels groups of mice (*p* = 0.3373). (**D**) Cocaine increased CCL2 levels and SF blocked such upregulation mediated by cocaine (* *p* < 0.05, vs. sham + saline; # *p* < 0.05, SF + cocaine vs. sham + cocaine, n = 5). (**E**) Cocaine decreased IL10 levels (* *p* < 0.05, vs. sham + saline). (**F**) There was no difference in TGFβ levels across groups of mice (*p* = 0.8469).

Previous investigations indicate that upregulation of neuroinflammation is involved in cocaine-mediated behavioral changes, and that microglial inhibition could block cocaine-mediated hyperlocomotion [33,34]. Since the striatum is inherently linked to locomotor activity, we monitored neuroinflammation marker levels in the striatum across groups of mice. The mice were sacrificed one day after the last cocaine injection and total RNAs were extracted from the striatum. The levels of pro-inflammatory mediators IL1β, IL6, TNFα, CCL2, and anti-inflammatory factors IL10 and TGFβ was assessed by qRT-PCR. Our results showed that cocaine significantly increased IL1β levels compared to sham + saline group and that SF reversed the upregulation effects [Figure 2A, one-way ANOVA, F (3, 20) = 12.67, *p* < 0.001]. However, there were no differences in the levels of IL6 and TNFα among the four groups [Figure 2B, one-way ANOVA, F (3, 20) = 1.082, *p* = 0.3794; Figure 2C, one-way ANOVA, F (3, 20) = 1.194, *p* = 0.3373]. Cocaine significantly increased CCL2 levels compared to sham + saline group, and this upregulation was reversed by SF [Figure 2D, one-way ANOVA, F (3, 8) = 6.908, *p* = 0.013]. For the anti-inflammatory factor IL10, cocaine significantly decreased its levels and the downregulation was partially reversed by SF [Figure 2E, one-way ANOVA, F (3, 17) = 5.725, *p* = 0.007]. There were no differences in TGFβ levels across groups [Figure 2F, one-way ANOVA, F (3, 8) = 0.2678, *p* = 0.8469]. Overall, cocaine significantly increased neuroinflammation marker levels by increasing the levels of IL1β and CCL2 and decreasing IL10 levels in the striatum and SF blocked the upregulations by modulating the effects of cocaine on the cytokines. Although previous studies showed that SDs could increase neuroinflammation in the hippocampus [28], we did not find that SF, by itself, had a significant impact on neuroinflammation in the striatum.

### 3.3. The Combined Effects of SF and Cocaine on Microglial Immune Responses

**Figure 3:**
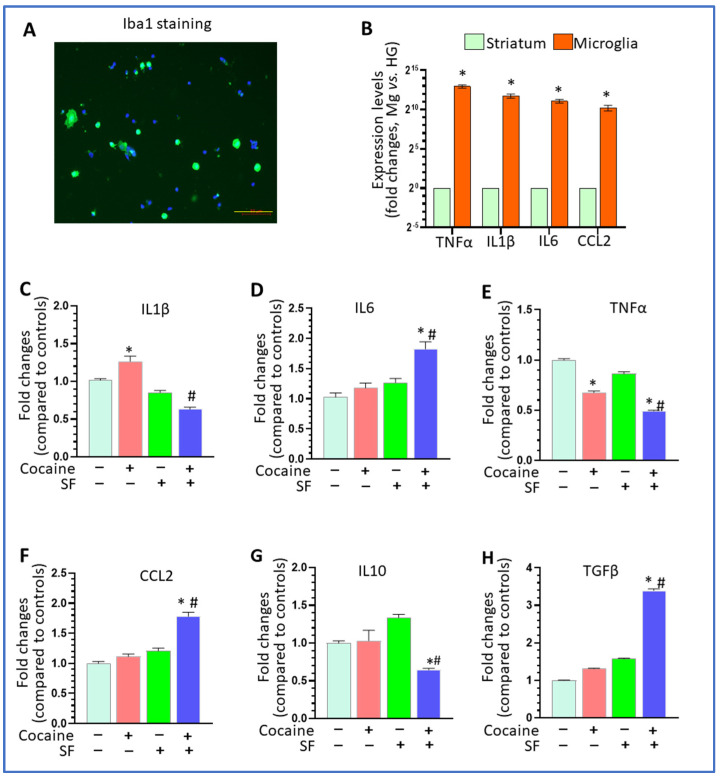
Combined effects of SF and cocaine on microglial immune responses. (**A**) Representative images for Iba1 immunostaining for adult microglia isolated from the striatum (scale bar = 50 μm). (**B**) The enrichment fold of TNFα, IL1β, IL6, and CCL2 in adult microglia comparing to striatal homogenates. (**C**) Cocaine increased IL1β levels in purified adult microglia and SF blocked upregulation mediated by cocaine (* *p* < 0.05, vs. sham + saline; # *p* < 0.05, SF + cocaine vs. sham + cocaine, n = 5). (**D**) Cocaine and chronic SF together significantly increased IL6 levels in purified adult microglia (* *p* < 0.05, vs. sham + saline; # *p* < 0.05, SF + cocaine vs. sham + cocaine, n = 5). (**E**) Cocaine and SF decreased TNFα levels in purified adult microglia (* *p* < 0.05, vs. sham + saline; # *p* < 0.05, SF + cocaine vs. sham + cocaine, n = 5). (**F**) Cocaine and chronic SF together increase SDs CCL2 levels in purified adult microglia (* *p* < 0.05, vs. sham + saline; # *p* < 0.05, SF + cocaine vs. sham + cocaine, n = 5). (**G**) Cocaine and SF together decreased IL10 levels in purified adult microglia (* *p* < 0.05, vs. sham + saline; # *p* < 0.05, SF + cocaine vs. sham + cocaine, n = 5). (**H**) Cocaine and SF together increased TGFβ levels in purified adult microglia (* *p* < 0.05, vs. sham + saline; # *p* < 0.05, SF + cocaine vs. sham + cocaine, n = 5).

Microglia are immuno-competent cells in the brain and serve as the main resource for cytokines and chemokines production. We therefore explored the effects of SF directly on microglia in the context of cocaine administration. We first determined that >90% of purified cells were positive for Iba1 staining and that the enrichment folds of IL1β, IL6, TNFα, CCL2 in purified microglia are 3373 ± 584, 2149 ± 345, 7912 ± 1023, and 1184 ± 289, respectively, compared to striatal homogenates (Figure 3A,B). For IL1β, there was a significant difference among the four groups (Figure 3C, one-way ANOVA, F (3, 20) = 43.27, *p* < 0.001). While cocaine increased IL1β compared to controls (*p* = 0.016), SF blocked upregulation and brought IL1β levels down even lower than that of controls (*p* = 0.0053). There were also significant differences in the levels of IL6 and TNFα across the groups (one-way ANOVA, *p* < 0.05). While cocaine and SF individually had no effects (*p* > 0.05), together they significantly increased IL6 levels (Figure 3D, * *p* < 0.05, vs. sham + saline; # *p* < 0.05, SF + cocaine vs. sham + cocaine). Interestingly, cocaine decreased TNFα levels in purified microglia, and SF could further reduce it (Figure 3E, one-way ANOVA, F (3, 20) = 245.9, *p* < 0.001; * *p* < 0.05, vs. sham + saline; # *p* < 0.05, SF + cocaine vs. sham + cocaine). For CCL2, cocaine and SF individually had no effects on its levels but there was synergistic upregulation in the SF and cocaine group (Figure 3F, one-way ANOVA, F (3, 20) = 54.15, *p* < 0.001; * *p* < 0.05, vs. sham + saline; # *p* < 0.05, SF + cocaine vs. sham + cocaine). For anti-inflammatory factors, SF and cocaine together significantly decreased IL10 levels and increased TGFβ levels, respectively (Figure 3G, one-way ANOVA, F (3, 12) = 14.65, *p* < 0.001; *p < 0.05, vs. sham + saline; # *p* < 0.05, SF + cocaine vs. sham + cocaine; Figure 3H, one-way ANOVA, F (3, 12) = 1705, *p* < 0.001; *p < 0.05, vs. sham + saline; # *p* < 0.05, SF + cocaine vs. sham + cocaine). Overall, the effects of SF and cocaine were complex and regulation differed across individual microglial cytokines and chemokines. 

### 3.4. Combined Effects of SF and Cocaine on Astrocytes

**Figure 4:**
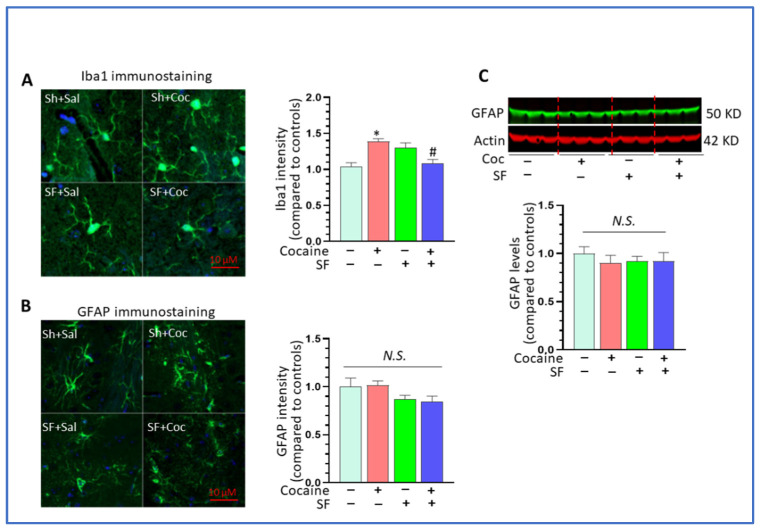
Combined effects of SF and cocaine on astrocytes. (**A**) Cocaine increased the signal intensity of Iba1 signaling and SF blocked its upregulation. Left: representative images for Iba1 immunostaining (scale bar 10 μM); right: statistical analysis for Iba1 signaling in these four groups of mice (* *p* < 0.05, vs. sham + saline; # *p* < 0.05, SF + cocaine vs. sham + cocaine, n = 5). (**B**) Cocaine and SF had no effects on the intensity of GFAP signaling. Left: representative images for GFAP immunostaining (scale bar 10 μM), right: statistical analysis for GFAP signaling in the four groups of mice (*p* > 0.05, n = 5). (**C**) Representative GFAP WBs image for the striatum across groups of mice; β-actin were served as a protein load control (*p* > 0.05).

We next explored the effects of SF and cocaine on astrocytes, another type of glia that contributes to neuroinflammation. An immunofluorescent approach was employed to analyze the intensity of Iba1 and GFAP in the striatum across groups of mice. The results showed that cocaine alone could significantly increase the intensity of the Iba1 signal and that SF could block its upregulation [Figure 4A, one-way ANOVA, F (3, 46) = 12.34, *p* < 0.001; * *p* < 0.05, vs. sham + saline; # *p* < 0.05, SF + cocaine vs. sham + cocaine]. These results were consistent with findings obtained by qRT-PCR. Interestingly, we did not observe a significant difference in GFAP levels across groups [Figure 4B, one-way analysis, F (3, 30) = 1.877, *p* = 0.145] and there was a trend toward downregulation in the SF and SF + cocaine groups. Also, the WBs results showed no significant difference in GFAP levels among groups (Figure 4C, one-way ANOVA, *p* > 0.05). Taken together, these data suggest that SF and cocaine mainly impacted microglia, not astrocytes, and that the altered neuroinflammation levels were mainly due to microglial dysregulation. 

### 3.5. Effects of Cocaine and SF on NLRP3 Inflammasome and Dopamine Signaling In Vivo

**Figure 5:**
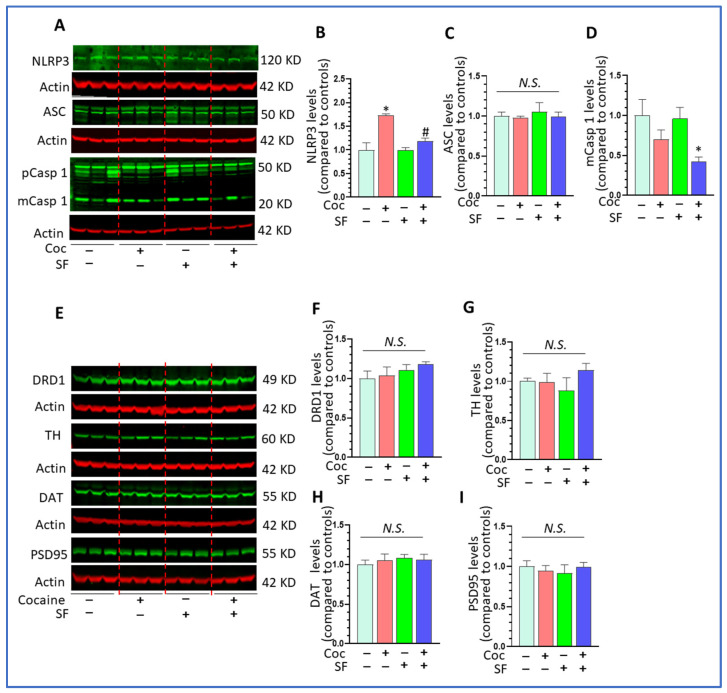
Effects of cocaine and SF on NLRP3 inflammasome and dopamine signaling in vivo. (**A**) Representative WBs images for NLRP3, ASC, and caspase 1 in the striatum across groups of mice; β-actin served as a protein load control. (**B**) NLRP3 levels in the striatum across groups of mice (* *p* < 0.05, vs. sham + saline; # *p* < 0.05, SF + cocaine vs. sham + cocaine, n = 5). (**C**) ASC levels in the striatum across groups of mice (*p* > 0.05). (**D**) mCasp 1 levels in the striatum across groups of mice (* *p* < 0.05, vs. sham + saline). (**E**) Representative WBs images for DRD1, TH, DAT, NLRP3, ASC, and PSDs95 in the striatum across groups of mice, β-actin served as a protein load control. (**F**–**I**) Levels of DRD1, TH, DAT, and PSDs95 in the striatum across groups of mice (*p* > 0.05).

SF mitigated cocaine-mediated upregulation of IL1β mRNA levels in the striatum and IL1β is the final executor for NOD-, LRR- and pyrin domain-containing protein 3 (NLRP3) inflammasome signaling [47,48] indicating that this neuroimmune pathway may participate in the effects of SF on cocaine-mediated locomotion. Therefore, we explored the effects of cocaine and SF on the NLRP3 inflammasome pathway in the striatum. Cocaine significantly increased the protein levels of NLRP3 and SF blocked upregulation (Figure 5A,B, one-way ANOVA, F (3, 6) = 9.318, *p* = 0.02). SF alone did not alter the levels of NLPR3 in the striatum. There were no differences in the levels of apoptosis-associated speck-like protein containing a CARD (ASC) among groups (Figure 5C, one-way ANOVA, *p* > 0.05). Interestingly, SF and cocaine together strikingly decreased the levels of mature (m) caspase 1 (Figure 5D, one-way ANOVA, *p* < 0.05, * *p* < 0.05 vs. sham + cocaine). These results suggest that SF could block cocaine-mediated upregulation in NLRP3 inflammasome activity. Locomotion activity is closely linked to striatal dopamine signaling and both cocaine and sleep disorders are capable of altering dopamine signaling [49,50]. Therefore, we determined the levels of four critical molecules regulating the dopamine system and the excitability of dopaminergic neurons across groups. Among these, DRD1 and DAT are critical for regulating the strength of dopamine signaling in the context of drug addiction [51], TH is a marker for dopaminergic neurons and PSDs95 levels represent the numbers of excitatory synapse in the brain [52]. Our results revealed no significant differences in the levels of these four proteins in the striatum indicating that there were no changes in dopamine signaling and neuronal excitability and that they were probably not involved in the effects of SF on cocaine-induced locomotor activity in our experiments (Figure 5E–I, one-way ANOVA, *p* > 0.05). 

### 3.6. Effects of SF on Cocaine-Mediated Locomotion Sensitivity and Neuroimmune Signaling in Withdrawn Mice

**Figure 6:**
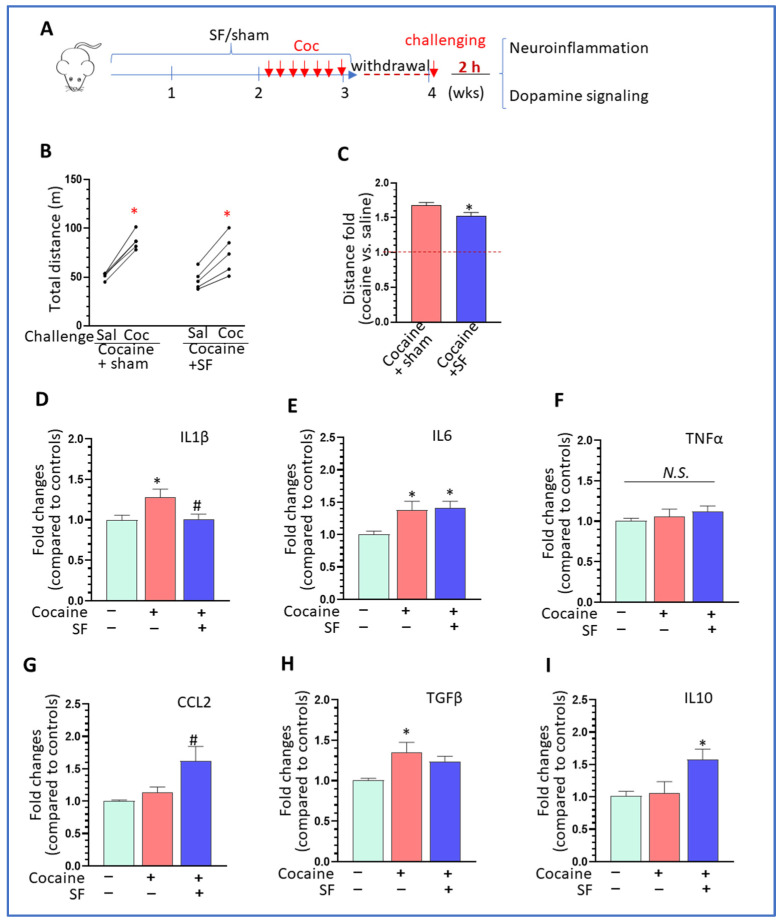
Effects of SF on cocaine-mediated locomotion sensitivity and neuroimmune signaling in withdrawn mice. (**A**) Schematic for the experimental design. (**B**) Challenging with cocaine significantly increased locomotor activity in both groups of mice (±SF, * *p* < 0.05). (**C**) The fold increase induced by cocaine in sham mice was significantly higher than in SF mice (* *p* < 0.05). (**D**) Challenging with cocaine significantly increased IL1β levels and SF blocked the upregulation (* *p* < 0.05, vs. sham + saline, # *p* < 0.05, vs. cocaine + sham). (**E**) Challenging with cocaine increased IL6 levels in both groups of mice (±SF) (* *p* < 0.05, vs. sham + saline). (**F**) There was no significant change in TNFα levels across the groups (*p* > 0.05). (**G**) Challenging with cocaine increased CCL2 levels in SF mice (* *p* < 0.05, vs. sham + saline). (**H**) Challenging with cocaine increased TGFβ levels in mice without SF but not in mice with SF (* *p* < 0.05, vs. sham + saline. (**I**) Challenging with cocaine increased IL10 levels in SF mice (* *p* < 0.05, vs. sham + saline).

Enhanced locomotion is believed to be a marker for psychoactive reward effects mediated by abused drugs after a period of withdrawal [53]. To explore the effects of SF on cocaine-mediated locomotion sensitivity, a separate cohort of mice that had been subjected to two weeks of SF and one week of cocaine (±SF + cocaine) were withdrawn from cocaine for one week. Then the mice received a cocaine (*i.p*., 10 mg/kg) challenge immediately followed by locomotion detection (Experimental Schematic shown in Figure 6A). As expected, the cocaine challenge significantly induced hyperlocomotion compared to saline injections in both groups (±SF) (Figure 6B, one-way ANOVA, *p* < 0.05) and the increase in activity induced by cocaine was significantly higher in the sham group than in the SF group (Figure 6C, two-way student-t test, * *p* < 0.05). We next determined levels of various cytokines in the striatum of withdrawn mice. IL1β was increased by cocaine challenge and again brought down by SF (Figure 6D, one-way ANOVA, F (2, 5) = 4.363, *p* = 0.0321). IL6 was increased in both sham + cocaine and SF + cocaine groups compared to the control (Figure 6E, one-way ANOVA, F (2, 5) = 5.297, *p* = 0.0182); however, there was no significant difference between the two treatment groups (±SF, *p* > 0.05). There was no difference in TNFα levels across groups (Figure 6F, one-way ANOVA analysis, F (2, 5) = 0.7665, *p* = 0.4820). There was no difference in CCL2 between sham + cocaine and control groups, but mice with SF and cocaine showed a significant increase (Figure 6G, one-way ANOVA analysis, F (2, 13) = 5.758, *p* = 0.0162). The anti-inflammatory factor, TGFβ, showed changes similar to IL1β, upregulation by cocaine and a decrease associated with SF (Figure 6H, one-way ANOVA, F (2, 15) = 5.021, *p* = 0. 0214). For IL10, there was no difference between sham + cocaine and control groups, but SF and cocaine together significantly increased its levels (Figure 6I, one-way ANOVA, F (2, 13) = 4.329, *p* = 0.0362). Overall, SF could partially block cocaine-induced locomotor sensitivity and differentially modulated the effects of cocaine on the levels of pro-, and anti- inflammatory factors in mice undergoing cocaine withdrawal followed by cocaine challenge. 

### 3.7. Effects of SF and Cocaine on Microglial Activation and Dopamine System in Withdrawn Mice

**Figure 7:**
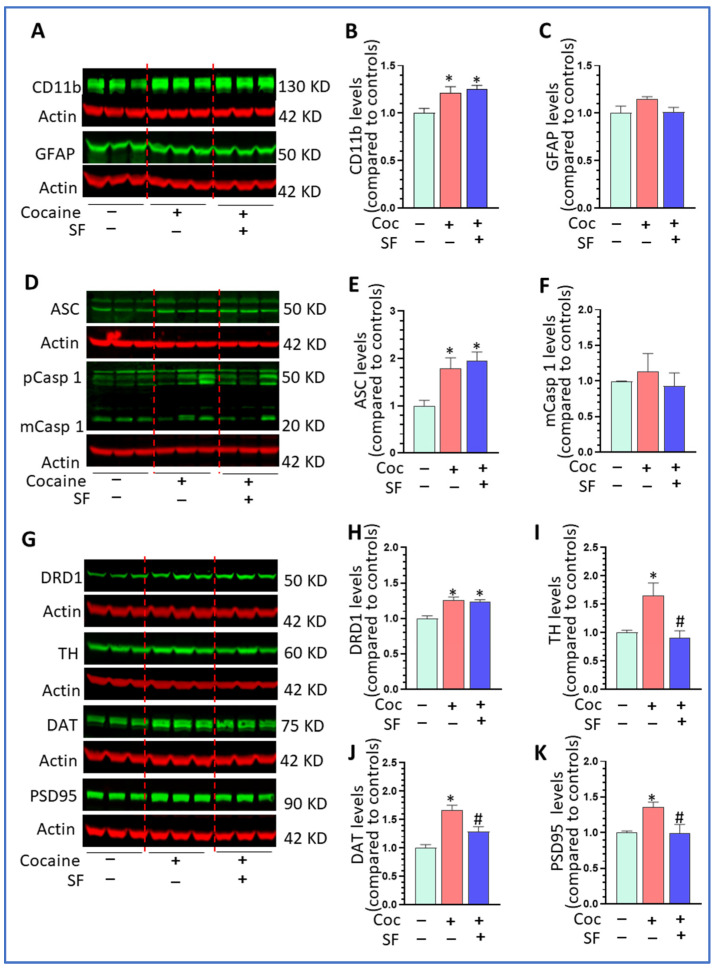
Effects of SF and cocaine on microglial activation and dopamine system in the withdrawn mice. (**A**) Representative WBs images for CD11b and GFAP in the striatum of three groups of mice; β-actin served as a protein load control. (**B**) Challenging with cocaine significantly increased CD11b levels in chronic ±SF mice (* *p* < 0.05, vs. sham + saline). (**C**) Challenging with cocaine had no effects on GFAP levels across groups (*p* > 0.05). (**D**) Representative WBs images for ASC and Casp 1 in the striatum of three groups of mice; β-actin served as a protein load control. (**E**) Challenging with cocaine significantly increased ASC levels in chronic ±SF mice (* *p* < 0.05, vs. sham + saline). (**F**) Challenging with cocaine had no effects on mCasp 1 levels across groups (*p* > 0.05). (**G**) Representative WBs images for DRD1, TH, DAT, and PSDs95 in the striatum of three groups of mice; β-actin served as a protein load control. (**H**) Challenging with cocaine significantly increased DRD1 levels in chronic ±SF mice (* *p* < 0.05, vs. sham + saline). (**I**) Challenging with cocaine significantly increased TH levels and SF blocked such upregulation (* *p* < 0.05, vs. sham + saline, # *p* < 0.05, vs. sham + cocaine). (**J**) Challenging with cocaine significantly increased DAT levels and SF blocked such upregulation (* *p* < 0.05, vs. sham + saline, # *p* < 0.05, vs. sham + cocaine). (**K**) Challenging with cocaine significantly increased PSDs95 levels and SF blocked such upregulation (* *p* < 0.05, vs. sham + saline, # *p* < 0.05, vs. sham + cocaine).

We next explored the levels of microglial activation status, NLRP3 inflammasome, and the dopamine system in withdrawn mice with cocaine challenge. Our results show that cocaine significantly increased CD11b levels independent of SF status (Figure 7A,B, one-way ANOVA, * *p* < 0.05 vs. sham + saline). No changes in GFAP levels were observed among groups of mice (Figure 7C, one-way ANOVA, *p* > 0.05). These outcomes again suggest that SF and cocaine mainly have effects on microglia and not on astrocytes. For the inflammasome pathway, although challenging with cocaine increased ASC in ±SF withdrawn mice compared to sham + saline mice (Figure 7E, one-way ANOVA, *p* < 0.05), there was no significant difference between the sham + cocaine and SF + cocaine groups (*p* > 0.05). In addition, there was no significant difference in mCasp 1 levels among the three groups (Figure 7F, one-way ANOVA, *p* > 0.05). For the dopamine system, while cocaine increased DRD1 levels in both mice groups with cocaine withdrawal (±SF), there was no difference between these two groups (Figure 7G,H, one-way ANOVA F (2, 6) = 13.49, *p* < 0.005). Cocaine significantly increased TH, DAT, and PSDs95 levels and SF blocked the effects (Figure 7I, one-way ANOVA, F (2, 6) = 13.49, *p* = 0.006; Figure 7J, one-way ANOVA, F (2, 6) = 17.24, *p* = 0.003; and Figure 7K, one-way ANOVA, F (2, 6) = 6.527, *p* = 0.03, respectively). These data revealed that SF could alter cocaine-mediate upregulation in the dopamine system in withdrawn mice. 

## 4. Discussion

SDs and drug addiction are major public health concerns around the world. They have high co-morbidity and their interactions can produce a vicious loop worsening the progression of each individual disease [5,6]. The harmful effects of abused drugs on sleep, including the time, quality, sleep-wake cycle, etc., have been well investigated; however, how SDs impact the development of drug addiction has been relatively underexplored, especially studies focused on underlying mechanisms. Our results show that even three weeks SF could alter cocaine-induced locomotor activity and sensitivity adding evidence that SDs could dysregulate the biological effects of cocaine. More investigations are needed to test the hypothesis that dysregulation of microglial neuroimmune signaling, and the dopamine system in the brain reward circuitry, could be responsible for the effects of SDs on the development of drug addiction. 

Several studies have reported on the effects of SDs on behaviors mediated by abused drugs. For example, acute sleep deprivation could potentiate or modulate cocaine-mediated locomotion in mice [42,54]. Sleep deprivation enhanced cocaine mediated CPP and impaired the extinction of cocaine-induced environmental conditioning in mice [55,56]. Sleep restriction increased the craving for cocaine in withdrawn rats at least partially through ionic glutamate receptors [57]. Acute total sleep deprivation potentiates amphetamine-induced locomotor and behavioral sensitization in mice [58], and it seemed that adolescent mice were more vulnerable than adults regarding sleep manipulations [59]. Sleep deprivation before extinction or reinstatement altered methamphetamine-mediated reward memory [60]. Intermittent rapid eye movement sleep deprivation attenuated the development of morphine tolerance and dependence in male rats [61], and chronic sleep deprivation blocked voluntary morphine consumption, but not CPP, in mice [62]. Sleep restricted rats showed increased alcohol consumption which was mediated by delta FosB induction [63] and resulted in complex effects on locomotion, rearing, and immobility behaviors [64]. In addition, acute total sleep restriction could enhance cue-induced reinstatement of alcohol seeking in male Wistar rats [65]. Overall, the effects of SDs on various types of abused drugs-mediated behavior are complex and can vary with the type and duration of sleep disruption, type of drugs, animal species, age, and behaviors assessed, with increases, decreases and no changes being reported across studies.

Most previous investigations of sleep and drug addiction utilized total sleep deprivation which does not model sleep problems typical in modern society, i.e., chronically reduced sleep time and quality. In our study, we used a mouse model of SF, which is a common SD that occurs with stress, aging and sleep apnea [66,67], and may mimic sleep problems humans often experience. Contrary to previous findings with acute sleep deprivation [42], our results showed that relatively longer SF reduced cocaine-mediated hyperlocomotion and sensitivity. The reduced locomotor activity was not likely associated with fatigue as there was no difference in locomotor activity between saline-treated controls and sham/SF mice. So, the contrasting results were probably due to the different regimens employed: acute (6 h) vs. three weeks of SF (12 h per day). Interestingly, we previously observed dysregulation in autophagy pathways in the striatum in mice with five days SF [68] but not after three weeks of SF (data not shown here). We also note that they [42] utilized Swiss male mice (three-month-old, 45–50 g, outbred) and we employed two-month-old C57/BL6 male mice (20–25 g, inbred), and that we cannot exclude the potential role of different mouse strains in the behavioral results. Another investigation showed that sleep deprivation decreased the locomotor response to acute cocaine but not to repeated cocaine in C57/BL6 male mice [54], the strain we used. However, these researchers did not observe differences in locomotion induced by cocaine challenge. Such discrepancy may be derived from the different cocaine dosages employed for the challenge. Our challenging cocaine dose was 10 mg/kg and theirs was 15 mg/kg. It is possible that the relatively higher dose of cocaine challenge could mask the mitigation effects of SF experience on hyperlocomotion. This possibility is also supported by the fact that a batch of mice with cocaine administered at a dose of 10 mg/kg in our studies revealed continuous mitigation effects of SF on locomotor activity. So, the dosage of cocaine used in experiments is another important factor that can affect behavioral results. The biological significance of the inhibitory effects of SF on abused drugs-mediated hyperlocomotion remains unknown. One possible hypothesis is that SF-experienced mice need greater amounts of abused drugs to activate microglia and neurons to reach the same level of behavioral changes that SF-naïve mice do. This could be related to the phenomenon of “drug tolerance”: the euphoric effects of abused drugs decrease after a certain period of administration [69]. Drug tolerance is known as a major contributor to the development of drug addiction. Our results suggest that ongoing SF or SF experience might contribute to the process of inducing drug tolerance, thereby promoting the likelihood of drug addiction. This possibility should be pursued in further investigations and using additional behavioral outcomes, such as self-administration. 

Previous studies have demonstrated that neuronal transmitters, such as dopamine, orexin, and glutamate, as well as circadian rhythms, are involved in mediating the effects of SDs on drug addiction [70,71,72,73,74], but the mechanisms remain largely unknown. Since SDs and abused drugs are both capable of inducing microglial activation, and because elevated neuroinflammation could promote both sleep disorders and drug addiction, we monitored neuroinflammation levels and microglial activation status in the brains of mice with various SF and cocaine treatments (±SF ±cocaine). We focused on the striatum which is a critical component of brain reward circuitry and also the most relevant for locomotor activity. Consistent with our previous publications [13,75], our results showed that cocaine significantly increased neuroinflammation levels, whereas SF partially blocked the increases. Also, SF modulated cocaine-mediated immune responses in adult microglia and the modulation varied across individual immune markers. The effects of cocaine and SF on IL1β mRNA levels were similar for both striatal and ex vivo isolated microglia. These results suggest that IL1β may be involved in the effects of SF on cocaine-mediated behaviors. Since the NLRP3 inflammasome pathway is the main source for mature IL1β production, we checked the levels of NLRP3, ASC and caspase 1 in the brains. Similarly, cocaine increased the levels of NLRP3 and SF blocked this upregulation. Mature caspase 1 was also decreased in the SF and cocaine groups. These findings strongly suggest that the NLRP3/IL1β axis is involved in the effects of SDs on cocaine-mediated locomotion. Our results also agree with previous findings demonstrating that IL1β is involved in cocaine-mediated locomotor activity in addition to the dopamine system [19,76]. Another interesting finding was that, with the exception of IL1β, the regulatory trends in other cytokines, including TNFα, IL6, and CCL2, TGFβ, were complex and different in striatal homogenates and in ex vivo isolated microglia. Microglia are believed to be the primary resource for cytokine and chemokine production and secretion in the brain; however, other types of brain cells, such as astrocytes, oligodendrocytes, endothelia, and even neurons and invading macrophages, could play roles in neuroinflammation. It is possible that the differences in the immune response between adult microglia and striatal homogenates in response to stimulus can be mainly derived by the complex composition of the homogenates, which includes almost every type of cells and microglia; yet, only accounts for about 10% of total cells. Such differences have been mentioned in previous investigations [77], which pointed out that microglial immune responses are often blunted by using brain homogenates and underscored the need for using purified microglia. Interestingly, we did not find any obvious changes in GFAP levels across the groups, suggesting that astrocytes are not extensively involved in neuroinflammation in our research paradigm. Other types of cells need to be examined in future studies. 

**Figure 8:**
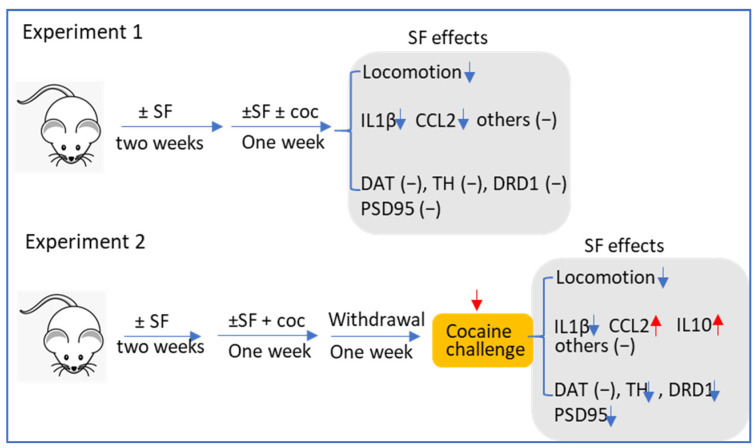
The summarization of the effects of SF on mice with different cocaine regimens.

In mice exposed to cocaine withdrawal followed by cocaine challenge, we observed significant microglial activation in both ±SF groups, as evidenced by elevated CD11b levels. Aside from IL1β, there were no differences in cytokine (TNFα and IL6) levels between ±SF groups, and CCL2 levels were even much higher in the SF and cocaine groups. Overall, it seemed that there was not much difference in neuroinflammation levels in mice exposed to cocaine withdrawal with or without SF, suggesting that neuroinflammation levels were not much involved in the effects of SF experience on cocaine-induced locomotor sensitivity. On the other hand, we did find that there were considerable changes in the dopamine system among groups: cocaine significantly increased the levels of TH, DAT, and PSDs95 and SF experience blocked such upregulation, which implied that previous SF history could block cocaine-mediated potentiation on dopamine system (neuronal excitability) and such blockage could affect cocaine-mediated locomotor sensitivity in mice undergoing cocaine withdrawal. It should also be noted that SF, as well as other SDs, can persist in humans for much longer than the three-week period we employed in our study, and that longer periods of SF and cocaine usage could have greater synergistic effects. In our studies, the modulation effects of SF on individual cytokines and chemokines and dopamine signaling in mice with different regimens are divergent. A side-by-side comparison of these two experiments is presented in Figure 8. The correlation between neuroinflammation (microglial activation) and psychostimulant-mediated behavioral changes has been well-addressed in previous reviews [24,25]. Here we found that SF could mitigate the hyperlocomotion stimulated by cocaine and that such behavioral regulation could be linked to microglial inhibition (the first experiment) and microglial inhibition and dopamine signaling (the second experiment). We do not exclude the possibility that other signaling pathways are also involved in the regulatory effects of SF on hyperlocomotion. We noticed that among all cytokines and chemokines that we investigated, only IL1β showed consistent results in both experiments. IL1β is the master gene regulating innate immune responses and numerous studies have demonstrated that IL1β is critical for microglial activation and that IL1β signaling could increase mesolimbic dopamine signaling [19]. Based on this prior work, we believe the IL1β downregulation could be linked to SF-mediated inhibitory effects on microglial activation and hyperlocomotion stimulated by cocaine. As for other proinflammatory mediators that did not show the same trend as IL1β, this might be attributed to the relatively low doses of cocaine we used and that IL1β may be more sensitive to cocaine exposure than other mediators. Actually, we observed the same upregulation trend in IL1β, IL6, and TNFα in the brains of mice with a dose of 20 mg/kg (manuscript in preparation). Overall, the differential responses from different cytokines and chemokines are probably due to the different regimens, the doses of abused drugs, as well as the time points for dissections. More investigations are needed to explore the causal relationship between neuroinflammation and drug addiction.

It is possible that the upregulation of dopamine signaling in experiment 2 (cocaine withdrawal mice with cocaine challenge) might also be due to cocaine withdrawal only. However, cocaine withdrawn mice showed significant upregulation of DAT and TH mRNA levels after cocaine challenge, compared to saline injection [78]. Based on this finding, we reasonably assumed that the upregulation on dopamine signaling in cocaine withdrawal mice was largely derived from cocaine challenge. The addition of another control group (withdrawal + saline) would help discern whether the upregulation of dopamine signaling came from withdrawal, this additional group will not change the main conclusions in our study but would be a valuable addition in future studies.

SDs have been demonstrated to activate microglia and have long-term effects on neuroinflammation [79,80]. In this study, we did not observe obvious microglial activation or increased neuroinflammation in mice with SF only. This discrepancy may arise from the different brain regions that were investigated. We focused on the striatum, while previous investigations emphasized either the hippocampus or prefrontal cortex. Indeed, we observed significant upregulation on neuroinflammation and microglial activation in the hippocampus of our mice (manuscript in preparation) and we hypothesize that the effects of SF on microglial activation and neuroinflammation is region-specific. As noted above, the duration of SF, and other SDs, may also be a factor in neuroinflammation.

In summary, the mechanisms underlying the interactions between drug addiction and SDs remain mostly unknown. Recently, accumulating evidence shows that microglia dysregulation (neuroinflammation) is inherently involved in these two brain diseases and might be the linkage bridging SDs and drug addiction. Microglial dysregulation is closely linked with neuropsychological symptoms, such as anxiety and depression [81,82], which are well-known negative factors contributing to drug craving and relapse during the withdrawal period. Also, the mutual effects of anxiety and depression and SDs have been well addressed [44,83], so targeting microglia may provide a promising approach to benefit both SDs and drug addiction.

## 5. Conclusions

Taken together, our results demonstrate that SF can reduce cocaine-induced hyperlocomotion and sensitivity which is involved in microglial neuroimmune signaling and the dopamine system. More investigations are needed to explore whether such dysregulations are responsible for the effects of SDs in promoting drug addiction. Since decreased activity in the reward network was found in chronic insomnia patients [84], our findings suggest that the mitigation effects of SF on cocaine-mediated microglial activation may underlie the linkage between SDs and drug addiction, and that targeting microglia may provide a promising approach to benefit both SDs and drug addiction. 

## Data Availability

Not applicable.
